# Protocol for the OUTREACH trial: a randomised trial comparing delivery of cancer systemic therapy in three different settings - patient's home, GP surgery and hospital day unit

**DOI:** 10.1186/1471-2407-11-467

**Published:** 2011-10-29

**Authors:** Pippa G Corrie, Margaret Moody, Victoria Wood, Linda Bavister, Toby Prevost, Richard A Parker, Ramon Sabes-Figuera, Paul McCrone, Helen Balsdon, Karen McKinnon, Brendan O'Sullivan, Ray S Tan, Stephen IG Barclay

**Affiliations:** 1 Oncology Centre, Addenbrooke's Hospital, Cambridge, UK; 2 West Suffolk Hospital, Bury St Edmunds, UK; 3 General Practice and Primary Care Research Unit, University of Cambridge, UK; 4 Centre for the Economics of Mental Health, Health Service and Population Research Department, Institute of Psychiatry, Kings College London, UK

**Keywords:** cancer treatment, chemotherapy, community care, care closer to home, out-patient service delivery

## Abstract

**Background:**

The national Cancer Reform Strategy recommends delivering care closer to home whenever possible. Cancer drug treatment has traditionally been administered to patients in specialist hospital-based facilities. Technological developments mean that nowadays, most treatment can be delivered in the out-patient setting. Increasing demand, care quality improvements and patient choice have stimulated interest in delivering some treatment to patients in the community, however, formal evaluation of delivering cancer treatment in different community settings is lacking. This randomised trial compares delivery of cancer treatment in the hospital with delivery in two different community settings: the patient's home and general practice (GP) surgeries.

**Methods/design:**

Patients due to receive a minimum 12 week course of standard intravenous cancer treatment at two hospitals in the Anglia Cancer Network are randomised on a 1:1:1 basis to receive treatment in the hospital day unit (control arm), or their own home, or their choice of one of three neighbouring GP surgeries. Overall patient care, treatment prescribing and clinical review is undertaken according to standard local practice. All treatment is dispensed by the local hospital pharmacy and treatment is delivered by the hospital chemotherapy nurses. At four time points during the 12 week study period, information is collected from patients, nursing staff, primary and secondary care teams to address the primary end point, patient-perceived benefits (using the emotional function domain of the EORTC QLQC30 patient questionnaire), as well as secondary end points: patient satisfaction, safety and health economics.

**Discussion:**

The Outreach trial is the first randomised controlled trial conducted which compares delivery of out-patient based intravenous cancer treatment in two different community settings with standard hospital based treatment. Results of this study may better inform all key stakeholders regarding potential costs and benefits of transferring clinical services from hospital to the community.

**Trial registration number:**

ISRCTN: ISRCTN66219681

## Background

In the last 10 years since the Calman-Hine report [1], cancer patient care in the United Kingdom has been reorganised to deliver treatment within cancer centres and cancer units within cancer service networks. Patients benefit by accessing specialised staff, equipment and treatment at the centre, while local cancer units provide treatment for common conditions, but the quality of care received is systematised across large geographical areas. During these years, cancer incidence has been rising in an ageing UK population, while developments in diagnosis and therapeutics alongside increasing patient expectations mean that more patients than ever before are being referred for cancer drug treatment. Oncology specialist services have adapted to meet increasing demand by delivering most drug treatments in purpose-built day units. However, capacity pressures are potentially driving down efficiency and risking maintenance of quality standards. The UK Government White Paper, 'Our health, our care, our say' [2], promotes patient choice and transfer of services closer to people's homes. Treatment for cancer patients in their own homes is well established in the private sector, but community treatment has been less extensively explored within the National Health Service. Few formal studies have been undertaken to evaluate the services that do exist.

Most reports in the literature have concentrated on evaluating delivery of cancer treatment in the home. These suggest that home care is safe, while patients may experience reduced side effects [3-5] and compliance rates may improve [6,7]. The cost of delivering treatment at home is poorly understood: some reports suggesting the possibility of cost savings [8-10], while others report home delivery to be more expensive [6,11]. Private providers are keen to work with the health service to deliver treatment in the community, but concerns regarding clinical governance and cost-effectiveness have been raised [12,13]. With few exceptions [12], most published studies are small and out-dated.

Community treatment models other than homecare already exist in the UK and include roving chemotherapy delivery buses as well as satellite units in community settings akin to models already established in other specialities such as cardiology and diabetes, where hospital teams visit GP surgeries on a regular basis. The Department of Health has given a clear steer to encourage commissioning of care closer to home despite a lack of quality data to justify this recommendation [14]. The OUTREACH study aims to address the patient benefits and costs associated with delivering treatment in two different community settings compared with standard hospital-based treatment, in order to better inform future commissioning decisions.

## Methods/design

### Study Hypothesis

The primary hypothesis is that community-based chemotherapy (whether at home or in a GP surgery) offers patients improved quality of care compared with standard hospital-based treatment: patient perceived benefit is thus the primary end point. The study aims to compare delivery of systemic cancer therapy in three different settings: hospital day unit, patient's home and Outreach GP surgery, by means of a randomised controlled trial.

### Primary Aim

Patient perceived benefit, as measured quantitatively by the emotional function domain of the EORTC QLQ-C30 quality of life questionnaire.

### Secondary Aims

1) Other patient benefits identified using the Hospital Anxiety and Depression score, EQ-5D (EuroQol) and a specifically designed patient satisfaction questionnaire.

2) Qualitative assessment of the experience of treatment delivered in the three different locations, using semi-structured interviews of patients, their carers (where appropriate), and other health care professionals (doctors, nurses and hospital managers either directly or indirectly associated with this study).

3) Impact on costs and cost-effectiveness of treatment delivered in the three different locations. It will evaluate whether community care is cost-effective by measuring the costs and benefits to the NHS and to patients.

4) Patient safety, determined by the number of adverse and serious adverse events.

5) Compliance with treatment delivered in the different locations.

6) Assessment of reasons why patients decline to take part in the study.

### Ethical approval

The protocol has research ethics committee approval from the Cambridgeshire 2 Research Ethics Committee and site specific assessment approval for each site involved in the study.

### Trial design and randomisation (Figure 1)

**Figure 1:**
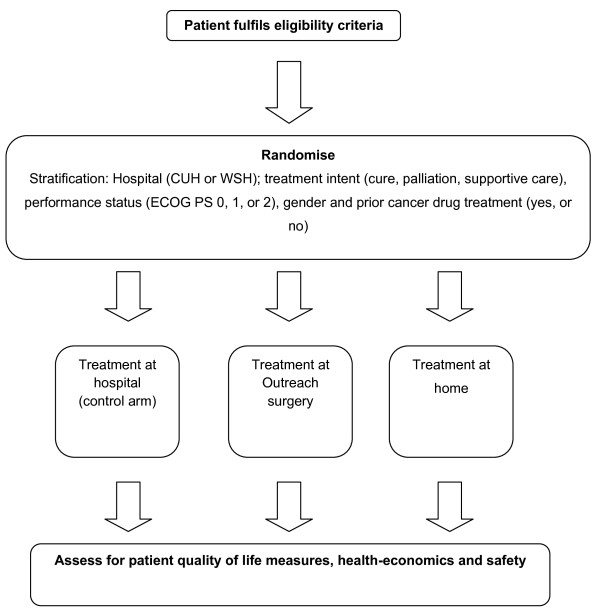
**Trial design**.

This is a prospective, randomised, controlled clinical trial conducted at two neighbouring Trusts in the Anglia Cancer Network: Addenbrooke's Hospital (CUH) and West Suffolk Hospital (WSH). Patients meeting the defined eligibility criteria are invited to take part in the study prior to or during their planned cancer treatment. All patients taking part provide written informed consent. Patients are randomised on a 1:1:1 basis to one of 3 arms: treatment delivered in the hospital out-patient and day unit environment (control arm A), treatment delivered at home (arm B) or treatment delivered in a GP surgery (arm C) using the method of minimisation with a random element to the assignment. Randomisation is performed centrally by statisticians located separately from trial recruitment and co-ordination and without knowledge of patients or their characteristics.

In the minimisation procedure the patients are stratified by centre (CUH or WSH), by treatment intent (cure, palliation in terms of disease control and life prolongation, or supportive care aimed at symptom control), ECOG performance status (PS 0, 1 or 2), gender, and prior cancer drug treatment (yes or no).

### Study duration

The study period is defined as being 12 weeks on a particular course of treatment. Where patients continue treatment beyond 12 weeks, those being treated at home can continue to do so. However, if they prefer to be treated in hospital they can transfer. Those patients randomised to hospital treatment cannot cross over to treatment in the community, since community-based treatment is not standard practice locally. Patients stopping treatment earlier than 12 weeks are not replaced in the study, but the reason for stopping is noted. They are included for the collection of study outcomes at 12 weeks, since analysis is on an intention-to-treat basis.

### Eligibility

Cancer patients over 18 years of age being treated at either CUH or WSH with standard infusions of under 4 hours duration and living within a 30 minute drive of either hospital will be offered entry to this study. Eligible patients must give written informed consent and be about to commence a course of treatment planned to last a minimum of 12 weeks, or have already commenced a course of treatment which is planned to continue for a minimum of 12 further weeks. The course of treatment may be aimed at cure, palliation (disease control and life prolongation) or supportive care (symptom control). Patients need to have hot and cold running water in the home, an indoor toilet and a working telephone. Patients must be ECOG performance status < 2, or if ECOG PS 2, there must be a second individual living in the home who functions as a carer.

Patients with life expectancy under 6 months or those dependent on hospital transport are excluded. Other exclusion criteria are: any patient receiving an unlicensed cancer drug treatment as part of a clinical trial where the drug is defined as an Investigational Medicinal Product unless the trial itself has received ethics and R&D approval to be conducted in designated community settings used in the Outreach trial: patients with language barriers or communication difficulties whose safety might potentially be comprised by entry into this trial: any patient where in the opinion of the Investigator entry into this trial would give cause for concern regarding patient or staff safety.

### Patient registration and randomisation

Eligible patients are registered with and randomised by the Outreach Trial Co-ordinator, at the Cambridge Cancer Trials Centre (CCTC), CUH. Eligible patients are allocated a unique trial number and the treatment location defined. Registration and randomisation is undertaken by fax or email and the randomisation outcome information provided to the Investigator by return, within 24 hours. The Investigator is responsible for organising the patient's treatment and follow-up.

### Study interventions

The Investigator has primary responsibility for the welfare of the patient for the duration of the study, whether receiving treatment in hospital, at home or in the GP surgery. Patient treatment is governed by a single set of standard operating procedures in use at CUH, WSH (and the GP surgeries involved in this study.

The Investigator is responsible for prescribing all treatment for trial patients, but can delegate this task to a member of his or her junior staff, as is standard clinical practice.

The treatment study period for this trial is 12 weeks, since most treatments are expected to be prescribed for at least this period of time. All planned treatment for a study patient will be delivered in the location to which the patient is randomised. Treatment of patients within the community may be aborted and transferred to hospital in the event of significant problematic drug-related toxicities, occurrence of a serious adverse event (SAE), on request by the patient, or following discussion between the community chemotherapy nurse and the Investigator who may feel it is in the patient's best interest to do so. The Investigator will review the patient in clinic prior to study entry and on completion of the study (whether at 12 weeks, or sooner in the case of early withdrawal). For patients continuing treatment beyond 12 weeks, the Investigator will review the patient on completion of the planned treatment or at 24 weeks, whichever is sooner.

All anticancer treatment and other prescribed drugs are dispensed by the oncology pharmacists in the respective hospitals, according to local policies. On the designated day, prescribed drugs for patients receiving treatment in the community are dispensed and placed with a copy of the patient prescription sheet in a designated box, ready for collection by the community chemotherapy nurse from the oncology pharmacy at a previously arranged time. The prescription sheet copy is returned to the oncology pharmacy within 48 hours of treatment.

### Treatment plan

The location of treatment delivery is decided at the point of trial entry. Once a patient had been randomised to a treatment location, the Investigator will define the treatment plan for the duration of the study period. Treatment will commence within 28 days of randomisation. A written summary of the treatment plan and emergency contact details will be provided to the patient. In all cases, the Investigator will prescribe treatment according to standard local practice.

For hospital-treated patients, all routine investigations and clinic appointments will be arranged according to standard clinical practice. For community-treated patients, the clinician will establish, with the community chemotherapy nurses, the treatment and follow-up arrangements for the patient for the duration of the study. This includes scheduled treatment times, need for any additional investigations and clinic appointments up until the end of the study period.

The chemotherapy nurses in hospital or the community will liaise with the Investigator to ensure the treatment plan for the patient is adhered to. They will document any deviations from the initial treatment plan and reasons for deviation. They liaise directly with the Investigator (or a member of his/her team) regarding any issues related to the patient's treatment. During the study, patients are asked to use the emergency contact details provided should problems of any kind arise.

### Study patient groups

#### Hospital (control) arm

Patients receiving treatment in hospital have their treatment delivered by appropriately trained chemotherapy nurses as per standard policies and procedures in each hospital.

#### GP surgery arm

Patients allocated treatment at a GP surgery are given the choice of being treated in one of 3 local surgeries in the area: The Christmas Maltings and Clements Surgery, Haverhill, East Barnwell Health Centre, Cambridge, or the Swan Surgery, Bury St Edmunds. All GP surgeries have generous, free parking facilities and their locations provide convenience for patients attending CUH and WSH. Patients entering this study do not have to be registered with these GP surgeries. All patients receive visual and written information regarding the geography of the surgeries as part of the invitation to take part in the trial to assist with their choice of surgery. Following allocation to this arm of the trial, a more detailed information leaflet and map describing the treatment venue is provided.

#### Home arm

Delivery of treatment in the home is delegated to trained chemotherapy nurses employed by CUH and WSH

### Patient assessments

Blood tests, radiological investigations and clinician reviews are undertaken on patients in all arms of the study, as per standard practice defined for each disease site and treatment regimen being used. Patients receiving treatment in the community have blood taken in the community and sent to the hospital laboratory so that the result is available to the Investigator on the day that treatment is due.

Prior to each course of treatment, patients receiving treatment in hospital are seen by the Investigator or delegated team member as per standard clinical practice, before proceeding with treatment. Patients receiving treatment in the community are assessed by the community chemotherapy nurse, who liaises with the prescribing clinician before proceeding with each course of treatment. Patients on any arm of the study can be referred for non-routine clinical review on request by another health care professional or on request by the patient. The frequency and reasons for extra clinical reviews will be monitored as part of the study, as part of adverse event reporting.

### Concomitant medication

Patients may receive all concomitant therapy deemed to provide adequate supportive care. Since this trial is not directly interested in treatment *per se*, concomitant medications will not be systematically recorded. However, information about ongoing treatment at the time of study entry and changes in treatment during the course of the study will be recorded by the patient in the Client Service Receipt Inventory (CSRI). In addition, records of drugs prescribed by the hospital and the patient's own GP will be obtained.

### Recording and collection of data

Upon randomisation of an individual patient, a pack containing specifically designed data collection forms, questionnaires and diaries is provided to the Investigator. The forms allow collection of essential information required for the study at each predefined trial data collection point. The study has four mandatory and 1 optional data collection point. Mandatory data collection points are baseline (at the start of study treatment), then approximately 4 weeks, 8 weeks and 12 weeks after commencing treatment. For patients continuing treatment beyond 12 weeks, there is an optional data collection point at 24 weeks or on stopping treatment, whichever occurs sooner. The baseline, 12 week and 24 week data collection visits take place at hospital. On these occasions, study forms will be completed by the Investigator or a member of his/her team, which may include a specialist or research nurse/practitioner. Patient questionnaires and diaries are given to the patient and/or collected from the patient as appropriate at these visits.

In order to allow for different treatment schedules, the second and third data collection points may be flexible to within 1 week either side of the planned 4 and 8 week time points. For patients having treatment in hospital, on these occasions, forms are completed by the Investigator or a member of his/her team, which may include a specialist or research nurse/practitioner. Patient questionnaires are given to and collected from the patient as appropriate at these visits. For patients having treatment in the community, these study forms are completed by the community chemotherapy nurse. Patient questionnaires are given to and collected from the patient as appropriate at these visits.

Following each planned data collection point, study forms and questionnaires are returned to the Outreach Trial Coordinator, who is responsible for creating and completing the unique patient Case Report Forms (CRF).

Patients randomised to receive treatment in either community setting may be transferred at any time to continue treatment in hospital, and the date and reason for transfer will be documented. Transfer of care to the hospital constitutes a serious adverse event (SAE) and withdrawal from the trial. Patients randomised to receive treatment in hospital may not be transferred to either location in the community, since this is not standard clinical practice locally.

### Study procedures

The study procedures are summarised in Table 1.

**Table 1:** Study measures and timing

	Data point 1: Baseline, prior to commencing treatment	Data point 2: 4** weeks into treatment	Data point 3: 8** weeks into treatment	Data point 4: 12 weeks into treatment	Data point 5: 24*** weeks into treatment
Clinic Review*	x			x	X

PS	x	x	x	x	X

Weight	x	x	x	x	

Blood tests*	x				

Radiology*	x				

EORTC QLQC30.	x	x	x	x	X

HAD, EQ-5D and Patient Satisfaction Questionnaire	x	x	x	x	X

CSRI Completion	x			x	

Patient Diary for AE Recording	x	x	x	x	X

Semi-Structured Interview	x(1 in 10 patients)			x	

*Clinic review, blood tests and radiological investigations will be carried out according to standard clinical practice at baseline and during the study period.

** +/- 1 Week

*** For patients whose treatment continues beyond 12 weeks, patients will complete a further set of trial assessments when their treatment stops or at 24 weeks, whichever is sooner. This data point is optional.

### Outcome measures

#### Study end points

##### 1) Primary end point: Patient perceived benefit

Patient perceived benefit is quantitatively measured by determining overall quality of life using the EORTC QLQC30 instrument. The mean quality of life emotional function score will be compared between study populations, since this domain might be considered to best reflect the levels of anxiety and satisfaction felt by patients taking part in this study.

The QLQC30 questionnaire will be administered prior to randomisation, at 4 weeks, 8 weeks and 12 weeks. Where patients continued to receive the same treatment beyond 12 weeks, they are asked to complete a similar questionnaire once more when their treatment stopped, or at 24 weeks, whichever is sooner. Where a carer is identified, the carer is also asked to complete EORTC QLQC30 questionnaire at each time point.

##### 2) Secondary end points

###### i) *Additional patient perceived benefits*

Additional comparisons of global quality of life, anxiety and overall satisfaction with treatment received are made by asking patients to complete three additional questionnaires, as follows:

- The EQ-5D (EuroQol) instrument

- The Hospital Anxiety and Depression (HAD) questionnaire

- A specifically designed patient satisfaction questionnaire

Each questionnaire is completed prior to randomisation, at 4 weeks, 8 weeks and 12 weeks. Where patients continue to receive the same treatment beyond 12 weeks, they are asked to complete a similar set of questionnaires when their treatment stopped or at 24 weeks, whichever is sooner. Where a carer is identified, the carer was also asked to complete the three questionnaires at each time point.

###### ii) *Semi-quantitative interviews*

To triangulate the quantitative patient data, prior to commencing treatment and after 12 weeks, one in every 10 patients (and main carer if available) is randomly selected in each arm, during the main randomisation, and asked to undertake a semi-structured interview to ask specifically about their experiences relating to the treatment received. Since the perceptions and experience of staff responsible for the service is also considered to be important, 12 clinicians responsible for patients entering the trial (at least 2 from WSH, the remainder from CUH), 1 doctors from each GP Surgery, 6 treatment delivery nurses (2 from WSH, 4 from CUH) and 2 senior level Oncology Managers at each hospital are being interviewed prior to and on completion of the study. All interviews are recorded, transcribed and subsequently analysed using a Framework approach.

###### iii) *Service use and cost data*

Service use and cost data relating to patients, the NHS and society as a whole, is collected and compared for all arms of the study. Use of the specific cancer drug treatments is centrally recorded. Other service use data was collected using an adapted version of the CSRI [15]. The CSRI has been used in around 200 health economic studies of health and social care interventions in the UK and internationally. Service use information is typically provided by the patient and/or family members (supplemented where possible with administrative data from providers) and the objective is to allow comprehensive costs to be generated.

By means of the adapted CSRI, patients are asked to provide information on a range of services used, taking place at Baseline and at 12 weeks. Services include contacts with staff directly related to the delivery of cancer care (oncologists, nursing staff, hospital-based pharmacists, etc), plus other healthcare professionals (GPs, primary care nurses, psychologists, etc), and social care professionals. Time spent in hospital (overnight stays plus day-patient attendances) is recorded. In addition, time per week spent by family/friends providing care (broken down into specific tasks) to the patient because of their condition is recorded.

A key cost is thought to be related to time spent travelling by patients and/or staff, to receive or deliver treatment. Patients are asked to state where contacts take place and time spent travelling to use them and also mode of travel. Many patients are accompanied and the CSRI also records information on this. A further section in the CSRI records time taken off work by patients and family members specifically because of the condition and treatment.

Data is also collected from the chemotherapy nurses. They are asked to document all scheduled and unscheduled contact with their patients as well as working hours relating to treatment of trial patients.

Unit costs are attached to the service use data to produce service costs for each patient in the study. The costs of delivering treatment in hospital, GP surgeries and at home are derived from information on salary costs, administrative and capital overheads, and drug costs. The unit costs of other services are obtained from nationally applicable data [16]. The cost of a home care worker is used to value care provided by family members/friends. Travel time costs are estimated using wage rates for those in employment and a proportion of this for those who are not in work.

Costs are compared between the three groups using bootstrap methods to account for the likely non-normal distribution of the data. Cost-effectiveness is assessed by combining service cost data with the primary outcome measure (QLQ-C30) and also Quality Adjusted Life Year (QALYs) generated from the EQ-5D [17]. Two-way comparisons are made between the groups. If one group has lower costs and better outcome over another then it would be dominant. If costs are higher and outcome better then incremental cost-effectiveness ratios would be constructed which will show the extra cost incurred to achieve an extra unit of outcome. To address uncertainty in the cost and outcome differences, cost-effectiveness acceptability curves are used which will show the probability that one option is more cost-effective than another for different values placed on a unit improvement in outcome.

###### iv) *Patient safety*

CUH and WSH have in place a comprehensive set of policies and procedures governing community-based care. Specifically for cancer, the Cancer Treatment in the Community (CTC) policy identifies low risk patients and low risk treatments usually offered to patients in the hospital out-patient and day unit facilities which are potentially suitable for delivery in the community (Table 1). In addition, the CTC policy describes a clinical governance framework for ensuring patient safety in the community, reflecting standard policies and procedures adhered to when delivering cancer treatment in hospital. To be considered for this trial, both the patients and the treatment being offered are required to meet the criteria listed in the CTC Policy.

The treatments given to patients taking part in this trial are by definition those which are well established, with an excellent safety record. Our hypothesis is that patients would not be adversely affected or harmed by receiving treatment in the community. Appropriate safety measures and procedures are in place for community treatment, to provide a safe environment considered to be equivalent to the hospital setting.

Patient safety will be evaluated by recording the number of adverse and serious adverse events which occur during the study period (see safety procedures).

###### v) *Compliance with treatment delivered in the different locations*

Patient compliance with their treatment is monitored as per standard local practice.

###### vi) *Non-participation in study*

The reasons why patients might decide not to take part in this study are of considerable interest. A short, anonymised questionnaire is offered to any patient who declines to take part. The patients are under no obligation to complete the questionnaire. Exploration of the responses on the returned forms offers insight into patient concerns regarding such delivery options, and therefore an indicator as to the likely take-up of future community chemotherapy delivery programmes.

In addition, all patients are monitored for symptom control, tumour response and overall survival according to standard clinical practice.

###### vii) Impact of treatment on local GP resources

Once a patient has completed the trial, a letter is sent to the patient's own GP requesting review of the patient notes held at the local GP surgery to document: the number of occasions the patient contacted his or her GP surgery during the trial period; what type of surgery staff were involved on each occasion (e.g. GP, nurse, other); the nature of those contacts, by classifying them as being related to a) cancer treatment, b) other need for treatment of any kind, c) support services, d) advice, or e) other. In addition, a summary print-out of all prescriptions is requested. The GPs receive a small remuneration for providing this information.

### Safety procedures

#### Adverse events

For the purposes of this trial, an adverse event (AE) is defined as:

- any unscheduled contact (e.g. phone calls, face-face visits etc) made by the patient, or carer to the outpatient or community treatment nurse, Investigator or member of his/her team, GP, or other support staff in relation to their current course of treatment.

- any unscheduled contact (e.g. phone calls, face-face visits etc) made by the out-patient, or community treatment nurse to the Investigator or member of his/her team, GP, or other support staff relating to the management of the patient receiving treatment.

To ensure accurate collection of trial-specific AEs, trial patients and treatment delivery nurses are issued with a purpose-designed diary in which to record all occasions when unscheduled contact is made. Specific information regarding contact is also requested. All AEs which occur from randomisation until the final trial visit are recorded. Specific information to be recorded in the diary regarding AEs includes the date and time of occurrence, main reason for making contact, with whom contact is made and outcome.

#### Serious adverse events

For the purposes of this trial, a serious adverse event (SAE) was defined as including any event which:

- results in death, regardless of cause.

- is life-threatening (immediate risk of death).

- requires hospitalisation or prolongation of existing hospitalisation, excluding those planned for cancer treatment, disease related procedures, or placement of an indwelling catheter, unless associated with other serious events.

- results in persistent or significant disability/incapacity.

- consists of a congenital abnormality of birth defect.

- involves grade 3 or 4 (severe or life threatening) drug-related toxicities.

- results in the patient's treatment being switched from the community to hospital-based treatment.

This trial is not evaluating treatment *per se*, but the venue of treatment delivery. Thus, reporting of drug-related AEs is limited to reporting of severe or life threatening toxicities, according to National Cancer Institute, Common Toxicity Criteria.

All SAEs which occur from randomisation until the final trial visit are recorded. All SAEs are reported to the CCTC immediately, by completing an SAE form, and assessed by the Chief Investigator. Autopsy data, where available, for deaths occurring from randomisation until 28 days after the last planned treatment administration are provided to the CCTC.

The relationship of SAEs to treatment delivery venue is assessed using the following definitions:

- Not related - The adverse event would definitely have occurred whatever the venue

- Unlikely to be related - The adverse event would probably have occurred whatever the venue

- Possibly related - The adverse event had a timely relationship to treatment venue. However, a potential alternative aetiology existed.

- Probably related - The adverse event had a timely relationship to treatment venue and a potential alternative aetiology was not apparent.

- Definitely related - The adverse event had a timely relationship to treatment venue and a potential alternative aetiology was not apparent. Upon switching treatment to hospital-based delivery, the adverse event would definitely not occur.

'Unlikely' and 'Not related' are considered not treatment venue related. 'Definitely', 'Probably' and 'Possibly' are considered treatment venue related.

In the case of an SAE, the subject will be followed up until recovery or stabilisation. The Investigator will take all measures necessary to resolve any SAE.

### Sample size

The EORTC QLQC30 questionnaire is a well validated instrument; this has been in widespread use since 1993 and is frequently used to assess quality of life of cancer patients in clinical trials. It comprises 30 questions representing various dimensions of quality of life. Of the 5 functional dimensions, emotional function might be considered to best reflect the levels of anxiety and satisfaction felt by patients taking part in this study. Using data on quality of life dimension mean scores determined by researchers interested in interpreting the results of the QLQC30 questionnaire, the standard deviation of the mean quality of life emotional function score for a large group of heterogeneous cancer patients is determined to be 24 [18,19].

With 130 patients randomised per arm, there is 80% power to detect a 10-point difference in mean QLQC30 score between any two study populations, at the 5% level of significance assuming a standard deviation 24. This allows for up to 30% of patients not providing information on the primary outcome, for reasons including withdrawal and non-completion of questionnaire. There is also 90% power to detect a 10-point difference between the home and GP surgery arms combined versus hospital using data from all three arms.

### Statistical Analysis

The primary analysis time-point is 12 weeks. The primary contrast is between home and GP surgery arms combined, versus hospital arm. Binary outcomes will be analysed using logistic regression. Continuous outcomes will be analysed using linear regression adjusting for randomisation stratifiers and the pre-randomisation baseline of the outcome in order to improve precision of the estimated intervention effects. Participants with valid data at follow-up but with missing data at baseline will be retained in the analysis by using the missing indicator method [20]. For measures repeated over time, linear mixed models will be used to analyse data from all time-points together, to assess for any patterns between study arms. The level of missing data will be compared by study arm. A logistic regression model will be used to examine whether baseline factors or intermediate study measures predict dropout by 12 weeks. With sufficient 12-week data, the impact of missing data on the main outcomes will be assessed using best and worst case sensitivity analyses, using multiple imputation [21], or otherwise using the linear mixed effects model with the available 4-week and 8-week data to model the 12-week effect from the trend in the outcome over time in each arm. Further analyses will include comparisons between pairs of arms, analyses at time-points and subgroup analyses, with subgroups defined by treatment intent and hospital.

### Trial organisation and management

The trial has a Trial Management Group (TMG), a Steering Committee and an independent data and safety monitoring committee (IDSMC). The TMG meets monthly. The IDSMC advises the Steering Committee regarding any evidence or reason why the study should be amended or terminated based on the recruitment rates or safety. The Steering Committee report their findings to the TMG.

The initial meeting of the ISDMC will take place 6 months after commencing recruitment, to assess safety of the study and recruitment efficiency. The IDSMC will then meet annually to monitor recruitment to the trial, protocol compliance as well as toxicity, serious adverse events and outcome.

## Discussion

Delivery of treatment in the community is of interest to the Department of Health, local commissioners and providers of health care, as well as to patients. Community cancer treatment service models are currently being established, but evidence of costs and benefits of these services is lacking. The Outreach trial is the first randomised trial undertaken in the UK to formally compare delivery of cancer treatment in the community with traditional hospital treatment. The results of this study will inform wider debate regarding implementation of these services in the future.

## Competing interests

The authors declare that they have no competing interests.

## Authors' contributions

PGC & AMM conceived this study. PGC, SIGB, ATP, PM, RS-F & AMM participated in the design and implementation of the study. VW, LB, HB, KM, RB, BO'S & RP participated in the implementation of the study. All authors participated in the preparation of the manuscript. All authors have read and approved the final manuscript.

## Pre-publication history

The pre-publication history for this paper can be accessed here:

http://www.biomedcentral.com/1471-2407/11/467/prepub
